# Structural analysis of cannabinoids against EGFR-TK leads a novel target against EGFR-driven cell lines

**DOI:** 10.1016/j.crphar.2022.100132

**Published:** 2022-09-30

**Authors:** Thomanai Lamtha, Lueacha Tabtimmai, Napat Songtawee, Natthasit Tansakul, Kiattawee Choowongkomon

**Affiliations:** a Laboratory of Protein Engineering and Bioinformatics (PROTEB), Department of Biochemistry, Faculty of Science, Kasetsart University, Bangkok, 10900, Thailand; b Spectroscopic and Sensing Devices Research Group (SSDRG), National Electronics and Computer Technology Center (NECTEC), National Science and Technology Development Agency (NSTDA), Pathumthani, 12120, Thailand; c Department of Biotechnology, Faculty of Applied Science, King Mongkut's University of Technology North Bangkok, Bangkok, 10800, Thailand; d Department of Clinical Chemistry, Faculty of Medical Technology, Mahidol University, Nakhon Pathom, 73170, Thailand; e Department of Pharmacology, Faculty of Veterinary Medicine, Kasetsart University, Bangkok, 10900, Thailand

**Keywords:** EGFR, Tyrosine-kinase inhibition, CBD, CBG, CBN, Molecular dynamics simulations, EGFR-TK, Epidermal growth factor receptor-tyrosine kinase, CBD, Cannabidiol, CBG, Cannabigerol, CBN, Cannabinol, SPR, Surface plasmon resonance

## Abstract

Epidermal growth factor receptor (EGFR) is a member of the ErbB family of proteins and are involved in downstream signal transduction, plays prominent roles in cell growth regulation, proliferation, and the differentiation of many cell types. They are correlated with the stage and severity of cancer. Therefore, EGFRs are targeted proteins for the design of new drugs to treat cancers that overexpress these proteins. Currently, several bioactive natural extracts are being studied for therapeutic purposes. Cannabis has been reported in many studies to have beneficial medicinal effects, such as anti-inflammatory, analgesic, antibacterial, and anti-inflammatory effects, and antitumor activity. However, it is unclear whether cannabinoids reduce intracellular signaling by inhibiting tyrosine kinase phosphorylation. In this study, cannabinoids (CBD, CBG, and CBN) were simulated for binding to the EGFR-intracellular domain to evaluate the binding energy and binding mode based on molecular docking simulation. The results showed that the binding site was almost always located at the kinase active site. In addition, the compounds were tested for binding affinity and demonstrated their ability to inhibit kinase enzymes. Furthermore, the compounds potently inhibited cellular survival and apoptosis induction in either of the EGFR-overexpressing cell lines.

## Highlights

• Cannabinoids reduced cell viability in EGFR-positive cells A431 and A549 by decreasing the tyrosine-kinase phosphorylation activity of EGFR.
• *In silico* analysis shows that cannabinoids bind to the active site of the EGFR-tyrosine kinase by the hydrophobic interaction and hydrogen bonding.
• CBD and CBG significantly induce cancer cells apoptosis in EGFR-positive cell A431.
• The consistent findings suggested that CBD and CBG could be developed as natural tumor-targeting agents for EGFR-positive cancers.

## Graphical abstract

**Figure:**
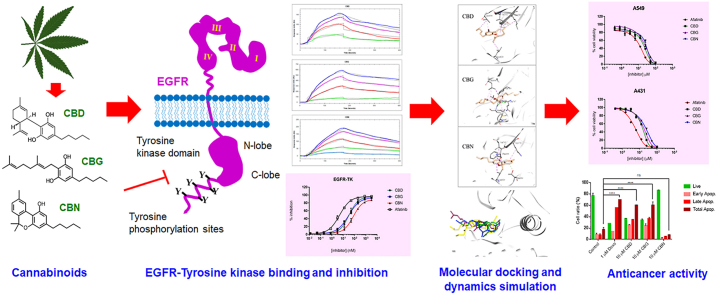

## 1 Introduction

Epidermal growth factor receptors (EGFRs; also known as ErbB1) are transmembrane glycoproteins with an intracellular tyrosine kinase and are members of the ErbB receptor family, with involvement in over 70% of all cancers. The ErbB receptor (homodimers and heterodimers), activated upon ligand binding, forms a signal transduction complex with several signaling proteins. Subsequently, at least five downstream signaling pathways (such as PI3K/Akt, Ras/ERK, and JAK2/STAT5) are activated, controlling cell proliferation, differentiation, and apoptosis (Bose, 2013; Kumagai et al., 2021; Roskoski, 2004; Yarden and Sliwkowski, 2001). More importantly, overexpressed EGFR and gene mutation has been reported in several human tumors of epithelial origin and is the target of multiple cancer therapies currently adopted in clinical practice (Turke et al., 2012; Yu and Hung, 2000).

Anti-EGFR treatments include monoclonal antibodies (mAbs), tyrosine kinase inhibitors (TKIs), immune therapies using vaccines, and antisense therapies. The anti-EGFR mAbs and TKIs are currently being evaluated in clinical trials. There are four major EGFR monoclonal antibodies approved for clinical usage, namely cetuximab, panitumumab, nimotuzumab, and necitumumab (Cai et al., 2020). However, several practical drawbacks for clinical use are apparent, including their large size and resistance to targeted therapy impairs clinical use and efficiency. EGFR tyrosine kinase inhibitors (EGFR-TKIs) have been introduced into the first-line treatment of non-small cell lung cancer (NSCLC) after the discovery of kinase-activating mutations of the EGFR gene (Nan et al., 2017). However, the drawbacks of this drug class are the financial burden to the patient and severe adverse reactions. If possible, an alternative therapy should be provided to patients at particular risk of known side effects of the TKI being prescribed (Thomson et al., 2022).

One of the world's oldest plant sources of medicines and textile fiber is *Cannabis sativa*, a member of the Cannabaceae family generally known as marijuana and a plant native to Central Asia. A mature cannabis plant contains more than 750 chemical compounds, of which over 100 various substances are classified as phytocannabinoids (Stefkov et al., 2022). Based on psychotropic properties, delta-9-trans-tetrahydrocannabinol (Δ9-THC) is the most studied, followed by cannabinol (CBN) and delta-8-trans-tetrahydrocannabinol (Δ8-THC). Of the non-psychotropics properties, the main research substances of interest are cannabidiol (CBD), cannabichromene (CBC), and cannabigerol (CBG). CBD is a major pharmacologically active phytocannabinoid, accounting for up to 40% of cannabis extract (Rong et al., 2017). The CBD shows therapeutic promise in the treatment of oxidative stress and inflammation-related conditions such cancer, metabolic, cardiovascular, and neurological disorders (Oguntibeju, 2019). Additionally, it has been noted that CBD causes many cancer cells to undergo programmed cell death. (Ligresti et al., 2006; Shrivastava et al., 2011). According to studies, CBD downregulate the metastatic factor (ID1) and up-regulates the pro-differentiation factor (ID2), which results in a significant reduction in the invasion glioblastoma (GBM), inhibition of GBM dispersal *ex vivo*, and reduction in tumor growth and Id-1 expression *in vivo* (Soroceanu et al., 2013). Interestingly, CBD inhibits breast cancer growth and metastasis through novel mechanisms by inhibiting EGF/EGFR signaling and modulating the tumor microenvironment (Elbaz et al., 2015). Furthermore, CBD promotes autophagy signal transduction via crosstalk between the ERK1/2 and AKT kinases, which represent potential regulators of cell proliferation and survival (Vrechi et al., 2021). However, the underlying mechanisms involved in these properties are still under discussion. As the direct precursor of CBD and THC, CBG, a minor cannabinoid, is present in the cannabis plant in very modest concentrations (less than 1%). It has been shown to be effective against breast cancer, be cytotoxic in high dosages to human epithelioid carcinoma cells, and to suppress keratinocyte proliferation (Baek et al., 1998; Ligresti et al., 2006; Pucci et al., 2013; Wilkinson and Williamson, 2007). Pharmacodynamic investigations have demonstrated that CBG interacts with the enzymes and receptors involved in carcinogenesis (Cascio et al., 2010; de Petrocellis et al., 2012). Additionally, CBG controls cell proliferation and differentiation by increasing DNA methylation on the keratin-10 gene, acting as a transcriptional repressor, which makes CBG a promising option for the development of novel skin disease therapies (Pedrazzi et al., 2021). Another study revealed that the combination of different cannabinoids, consisting of including TCH, CBG, CBN, and CBD, on human breast cancer cell lines can cause cell-cycle arrest in G2 phase following apoptosis without having any adverse cytotoxic effects on normal cells (Schoeman et al., 2020). CBN, a weak psychotropically active cannabinoid, is an oxidized degradation product of THC. It has been shown to possess anti-inflammatory, sedative, anticonvulsant activity. (Russo and Marcu, 2017). CBN acts independently on the CB1 and CB2 receptors and reportedly has TRPA1 agonistic and TPRM8 antagonistic effects (de Petrocellis et al., 2011). This compound has potentially inhibited keratinocyte proliferation (Wilkinson and Williamson, 2007).

All these compounds interact with a number of receptors, such as the cannabinoid receptors CB1 and CB2, to induce a variety of biological effects. However, there are other non-cannabinoid receptors, including TRPM8, G-protein coupled receptors (GPR55, GPR3), and ion channels. (Borrelli et al., 2014; Massi et al., 2013). Additionally, EGFR activation can occur through transactivation by other receptors and mediators (Block and Klarlund, 2008; Spix et al., 2007). Human corneal epithelial cell (HCEC) proliferation and migration are increased in response to CB1 and TRPV1 activation through EGFR transactivation, which stimulates the MAPK and Akt/PI-3K pathways globally. On the other hand, the TRPV1-mediated increases in IL-6 and IL-8 release are elicited through both the EGFR-dependent and EGFR-independent signaling pathways (Yang et al., 2010). However, the antitumor role of phytocannabinoids on tyrosine-kinase phosphorylation of EGFR-tyrosine kinase (EGFR-TK) are not well characterized.

In the present study, we demonstrated binding affinity and the inhibitory properties of CBD, CBG, and CBN on EGFR-tyrosine kinase, and *in silico* characterization of the molecular interaction between cannabinoids and EGFR-TK. Furthermore, we showed that CBD and CBG inhibit EGF-induced EGFR-TK activity and cause inhibition of epidermoid carcinoma A431 ​cell growth, which can lead to apoptotic death. Our findings suggest that CBD and CBG could be developed for EGFR TKIs-based treatment options in patients with EGFR-positive cancers.

## 2 Materials and methods

### 2.1 Compound preparation

**Fig. 1:**
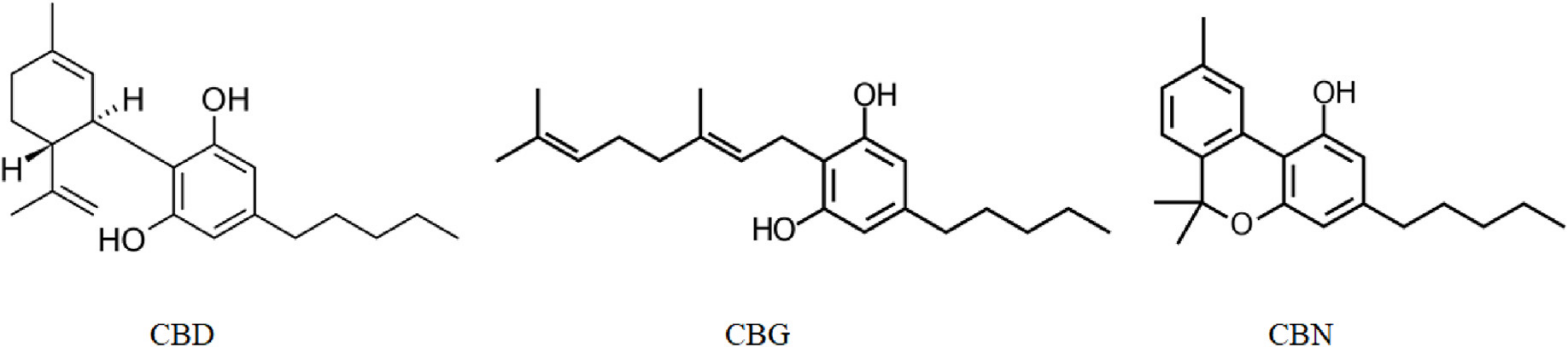
Molecular structure of cannabinoids used in the experiments.

Pure, isolated cannabinoids (CBD, CBG, and CBN, see Fig. 1) were purchased from Mile High Labs (Broomfield, USA). The compounds were prepared as stock solutions in dimethyl sulfoxide (DMSO) and stored at −80 ​^°^C until used. For the biological assay, the stock solutions were diluted in the culture medium or reaction buffer to the designated concentrations with the DMSO concentration not greater than 1% (v/v).

### 2.2 Expression and purification of EGFR-TK

The tyrosine kinase domain of EGFR was cloned into the pCold I vector (Addgene, USA). The protein was over-expressed in *E. coli* Rosetta™ (DE3) (Novagen, Germany) host cells induced by 0.5 ​mM IPTG overnight at 16 ​^°^C in Luria-Bertani medium. After cell lysis and centrifugation, the protein was purified from supernatant using a DEAE-Sepharose column (Cytiva™, USA). The most approximate molecular weight fraction was dialyzed against dialysis buffer at 4 ​^°^C overnight. Then, the dialysate was applied to a Resource™ Q column (16 ​mm diameter ​× ​30 ​mm bed height, Cytiva™, USA), connected to an FPLC System (GE ÄKTA FPLC™, USA), equilibrated with dialysis buffer (pH 7.4), and eluted using a linear gradient of 0–30% elution buffer. The protein was finally polished using a Superdex™ 200 ​pg column (Sigma-Aldrich, USA) with PBS (pH 7.4).

### 2.3 Kinase inhibition assay

The inhibitory activity of the pure cannabinoids (depicted in Fig. 1) against EGFR-TK was examined using the ADP-Glo™ Kinase Assay (Promega, USA). The manufacturer's recommended procedure was followed during the experimental process. The reaction mixture (25 ​μL) contained 5 ​μL of kinase buffer (20 ​mM Tris-HCl (pH 7.5), 20 ​mM MgCl_2_, 0.1 ​mg/mL BSA), 5 ​μL of 25 ​μM ATP, 5 ​μL of 12.5 ​μg/mL poly (Glu:Tyr) substrate, 5 ​μL of 1 ​ng/μL EGFR-TK, and 5 ​μL of various compound concentrations. Lapatinib and Afatinib (NCI, USA), were used as control kinase-inhibitor. The reactions took place in a 384-well plate (solid white, Greiner Bio-One Lumitrac plate, Austria), and incubated at room temperature for 1 ​h. The kinase reaction was then stopped, and the leftover ATP was depleted at room temperature for 40 ​min, using 5 ​μL of the ADP-Glo reagent. After that, 10 ​μL of ADP-Glo detection agent was added for 30 ​min in order to simultaneously convert ADP to ATP and enable the measurement of the newly synthesized ATP utilizing a luciferase/luciferin reaction. Luminescence measurements were made using a microplate spectrophotometer (Synergy HTX Multi-Mode reader, BioTek, USA). The half-maximal inhibitory concentration (IC_50_) values of inhibitors were determined using the nonlinear regression analysis of the log test compound concentration versus percentage control activity plots in the GraphPad Prism program (GraphPad Software Inc., USA). All assays were done in triplicate.

### 2.4 Estimation of compound binding affinity using surface plasmon resonance (SPR)

The binding affinity of cannabinoids to EGFR-TK were performed on an Open SPR 2-Channel Starter Pack R4.2 (Nicoya, Canada) at 25 ​°C. The compounds were injected into the flow-channel at various concentrations (0–50 ​μM) and subsequently passed over the EGFR-TK-immobilized Amine Sensor Chip (NECTEC, NSTDA, Thailand). Briefly, the EDC/NHS coupling reaction, which consisted equal volumes of 0.4 ​M EDC and 0.1 ​M NHS were combined; the EDC/NHS mixture was then combined with EGFR-TK (100 ​μg/mL) at a ratio of 1:1 and incubated for hour on ice. The carboxyl groups were activated with EDC/NHS, which forms an NHS ester and allows for efficient conjugation to primary amines to form an amide bond at physiologic pH. The activated EGFR-TK was immobilized by amine-coupling chemistry on the sensor chip at 4000 response units at a flow rate of 20 ​μL/min in running buffer (sterile filtered, and degassed PBS buffer (pH 7.4)). The surface was subsequently deactivated by a 10 ​min injection of 1 ​M ethanolamine-HCl pH 8.5 (Sigma-Aldrich, USA) to block the remaining active carboxyl groups and reduce non-specific binding. To determine binding kinetics, the compounds were flowed at 30 ​μL/min over the chip surface at various concentrations (6.25, 12.5, 25, and 50 ​μM) in PBS buffer (pH 7.4) containing 5% DMSO. The sensorgrams were examined using the Trace Drawer 1.9.1 software (Ridgeview Instruments, Sweden).

### 2.5 Molecular docking

A crystal structure of the EGFR-TK domain in complex with the FDA-approved drug Erlotinib was obtained from the PDB entry 1M17 (Stamos et al., 2002). Its extended C-terminal tail (Leu977–Pro995), water, and Erlotinib molecules, as well as the alternative coordinates of Cys751 and Asp831 were removed from the structure. The structural model of the 1M17-based EGFR-TK domain, which includes the juxtamembrane portion (Gly672–Ile682), core TK domain (Leu683–Leu955), and C-terminal portion (Val956–His964) was used as the receptor. The atomic coordinates of the three cannabinoids (CBD, CBG, and CBN) were obtained from PubChem CID 644019, 5315659, and 2543, respectively. GOLD 5.6 (Cole et al., 2005; Cullen et al., 2015; Jones et al., 1997; Verdonk et al., 2003) was used to conduct cannabinoid molecular docking against EGFR-TK. The docking site was defined by the amino acid residues within a radius of 10 ​Å of Erlotinib molecules (namely AQ4). The Kinase ChemScore (KCS) Fitness Function was used to evaluate molecular docking, which works well for protein kinase models when compared to other scoring functions. (Sawatdichaikul et al., 2012). For each compound, one hundred conformational poses were generated. Finally, the binding energy and fitness score were calculated, ranked, and then used to find the best pose for each cannabinoid complexed with EGFR-TK by the GOLD CCDC module.

### 2.6 Molecular dynamics simulation

All simulations were run with explicit-solvent periodic boundary conditions using GROMACS 2020.1 (van der Spoel et al., 2005). The three simulation models included EGFR-TK bound to each of the docked cannabinoid structures, which are referred to here as *(i)* EGFR-TK/CBD, *(ii)* EGFR-TK/CBG, and *(iii)* EGFR-TK/CBN. Prior to a simulation setup, the N- and C-termini of the EGFR-TK model's peptide chain were capped with the acetyl and amino-methyl groups, respectively.

The AMBER99SB-ILDN force field (van der Spoel et al., 2005) was applied in all simulation models for EGFR-TK structural analysis, and the ionization state of the amino acid structures was set according to a standard protocol. Partial atomic charges of all three cannabinoid structures were estimated according to the semi-empirical AM1-BCC model, which were parameterized to reproduce the HF/6–31G∗ restrained electrostatic potential charges (Jakalian et al., 2002). Then, GROMACS topologies for CBD, CBG, and CBN were generated on the ACPYPE server (Sousa da Silva and Vranken, 2012). Each simulation model was solvated in a rectangular box of TIP3P water molecules (Jorgensen et al., 1983). Keeping 1.2 ​nm between the solutes and the sides of the solvent box. One chloride molecule was randomly added to neutralize the charge in the system. The energy of all the solvated systems was then minimized using the steepest descent algorithm, either until the maximum force on any atom was less than 1 ​kJ ​mol^−1^∙nm^−1^ or until additional steps resulted in a potential energy change of less than 1 ​kJ ​mol^−1^.

The simulation systems were equilibrated in two phases with position restraint (at the force constant of 1000 ​kJ ​mol^−1^∙nm^−1^) applied to all heavy atoms of the protein and inhibitors, allowing hydrogen atoms and water and ion molecules to move freely. The first phase was conducted under NVT conditions at 300 ​K using a modified Berendsen velocity rescaling thermostat (Bussi et al., 2007). The second phase was then carried out under NPT conditions at 1 ​bar of pressure with a Parrinello-Rahman barostat (Nosé and Klein, 1983). After equilibration, any position restraint for any of the heavy atoms in the systems was gradually removed. Subsequently, 100 ns unrestrained dynamics production was performed under the NPT conditions at 300 ​K and 1 ​bar of the system. Bond lengths were constrained for all dynamics processes using the LINCS algorithm, which allows for a 2.0 fs time step (Hess et al., 1997). For both electrostatic and van der Waals interactions, the cut-off distance for the short-range neighbor list was set at 1.2 ​nm. Long-range electrostatic interactions were approximated using the PME method (Darden et al., 1993). The atomic coordinates were recorded every 10 ps for data collection and analysis. Intermolecular interactions were observed and illustrated using the Discovery Studio package (Stierand and Rarey, 2010).

The binding free energy of EGFR-TK with cannabinoids was calculated using the molecular mechanics Poisson-Boltzmann surface area (MM-PBSA) method (Baker et al., 2001) with the command *g_mmpbsa* (Kumari et al., 2014), with calculations to obtain the minimized-docked and average simulated complex structures of each trajectory. In addition, the binding energies were decomposed per residue to examine the individual energy contributions of each residue to the enzyme-inhibitor interaction.

### 2.7 Cell culture

NIH/3T3 (mouse fibroblast), A549 (EGFR-positive human lung cancer), and A431 (EGFR-positive human epidermoid carcinoma) were purchased from Biomedia (Thailand) Co., Ltd. The cells were grown in growth medium (Dulbecco's Modified Eagle Medium, DMEM, Gibco, USA) containing 10% fetal bovine serum (ATCC, USA) and 100 U/mL antibiotics (Sigma-Aldrich, USA) in plastic tissue culture dishes as adherent monolayers. Before setting up the experiment, the cells were maintained at 37 ​°C in a humidified incubator with a 5% CO_2_ environment, and they were replenished every 3 days.

### 2.8 Cell viability assay

Anticancer activities against different cancer cell lines were evaluated using the MTT assay based on the reduction of the tetrazolium dye to insoluble formazan by live cells. One normal and two cancer cell lines with EGFR overexpression (NIH/3T3, A549, and A431, respectively) were seeded at a density of 5 ​× ​10^3^ ​cells per well in 96-well plates and incubated for 16 ​h at 37 ​°C with 5% CO_2_, then treated with 100 ​μL of various concentrations of the samples and incubated for 72 ​h. Anticancer chemotherapeutic drugs, Afatinib and Lapatinib (Santa Cruz Biotechnology, USA) was used as reference compound. After incubation, the culture medium was replaced with 100 ​μL of MTT (Thermo Fisher Scientific, USA) solution (0.5 ​mg MTT in 1 ​mL culture medium) and incubated at 37 ​^°^C for 3 ​h. The medium was then removed and 50 ​μL of DMSO was added to each well for solubilization of formazan. The absorbance of each well was measured at 570 ​nm using a microplate spectrophotometer (Synergy HTX Multi-Mode reader, BioTek, USA). IC_50_ values were calculated by comparing the absorbance of compound-treated wells to the untreated control, using the GraphPad Prism7 software (GraphPad Software Inc., USA). All experiments were done in triplicate.

### 2.9 Apoptosis assay

To determine the apoptotic ratio of the cells treated with cannabinoids, flow cytometric analysis with annexin V was used to determine the cellular state. A431 was seeded at a density of 5 ​× ​10^4^ ​cells per well in 24-well plates and incubated for 16 ​h at 37 ​°C with 5% CO_2_. The cells were treated for 72 ​h with or without doxorubicin (1 ​μM) and cannabinoids (10 ​μM). The cells were taken out of the medium containing the compounds, washed with PBS, harvested with trypsin, and then resuspended in 100 ​μL of medium before being transferred into a 1.5 ​mL tube. Then, 100 ​μL of Muse®™ Annexin V & Dead Cell reagent (Merck Millipore, USA) was properly mixed with the cellular samples before incubating them for 20 ​min at room temperature in the dark. Finally, the samples were then run in Annexin V & Dead Cell mode on a Muse®™ Cell Analyzer (Merck KGaA, Germany). In order to discriminate between non-apoptotic, early apoptotic, late stage apoptotic, and dead cell populations, data was generated using the Muse System, which offers statistical values indicating the percentage of healthy and apoptotic cells.

### 2.10 Statistical analysis

All experiments were performed three times and the differences between the experimental and control cells were analyzed based on Student's t-test. Data were presented as means ​± ​SD of three independent experiments and P ​< ​0.01 indicated a significant result.

## 3 Results

### *3.1* *In vitro* tyrosine kinase inhibitory activity of cannabinoids

**Fig. 2:**
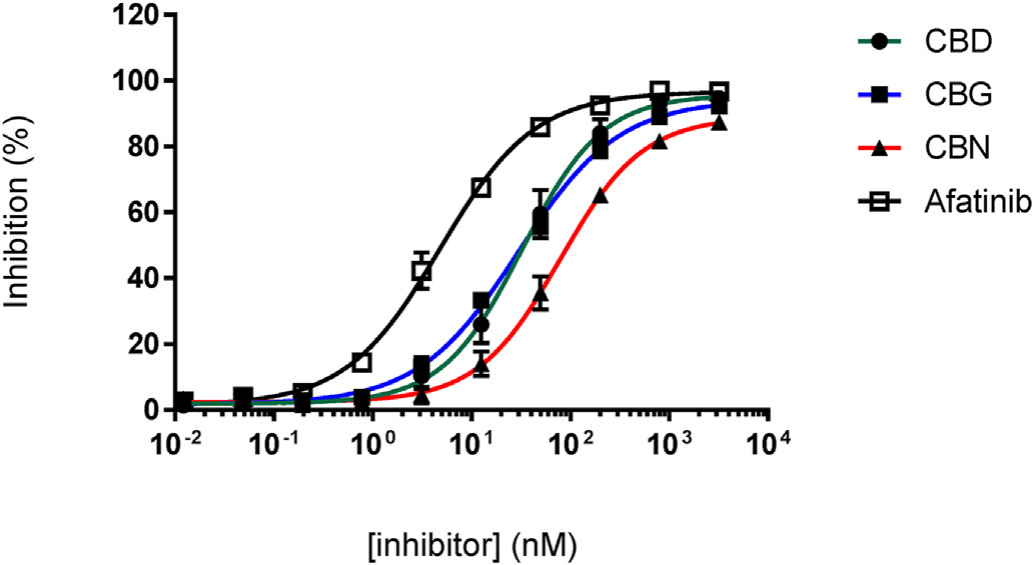
*In vitro* kinase inhibitory activity of cannabinoids and Afatinib against EGFR-TK. Graphs were plotted as percentage inhibition of kinase activity versus log of dose, estimated using GraphPad Prism (GraphPad Software Inc., USA). IC_50_ values are given in Table 1, results are expressed as mean ​± ​SD of triplicate experiments.

The studied compounds (shown in Fig. 1) were tested for their kinase inhibitory activity. The behavior of CBD and CBG demonstrated the potent inhibitory effect against EGFR-TK with IC_50_ values of 32 ​nM and 29 ​nM, respectively, whereas CBN shown less activity (Fig. 2 and Table 1). Compared to CBN, CBD and CBG were more effective at inhibiting kinases. However, these compounds need to be further evaluated for their binding affinity to EGFR-TK and growth inhibition effects on EGFR-positive cancers.

**Table 1:** Summary of IC_50_ values for *in vitro* kinase inhibitory activity against EGFR-TK and anticancer activity against cancer cells of cannabinoids.

Compounds	EGFR-TK inhibition IC_50_ (nM)	Growth inhibition IC_50_ (μM)		
		NIH/3T3 (EGFR-)	A549 (EGFR+)	A431 (EGFR+++)
Afatinib	3.37 ​± ​3.13	11.29 ​± ​3.39	12.76 ​± ​3.90	5.039 ​± ​2.56
CBD	32.63 ​± ​3.16	238.6 ​± ​2.00	22.44 ​± ​4.75	14.18 ​± ​3.41
CBG	29.9 ​± ​2.10	124.4 ​± ​1.90	29.43 ​± ​2.13	17.65 ​± ​2.88
CBN	78.79 ​± ​2.20	116.4 ​± ​2.35	30.45 ​± ​6.99	28.42 ​± ​2.33

Values are mean ​± ​standard deviation (SD); EGFR-, EGFR-negative; EGFR+, 1–2 ​× ​10^5^ EGFR/cell; EGFR+++, >4 ​× ​10^5^ EGFR/cell.

### 3.2 Binding affinity of cannabinoids to EGFR-TK

**Fig. 3:**
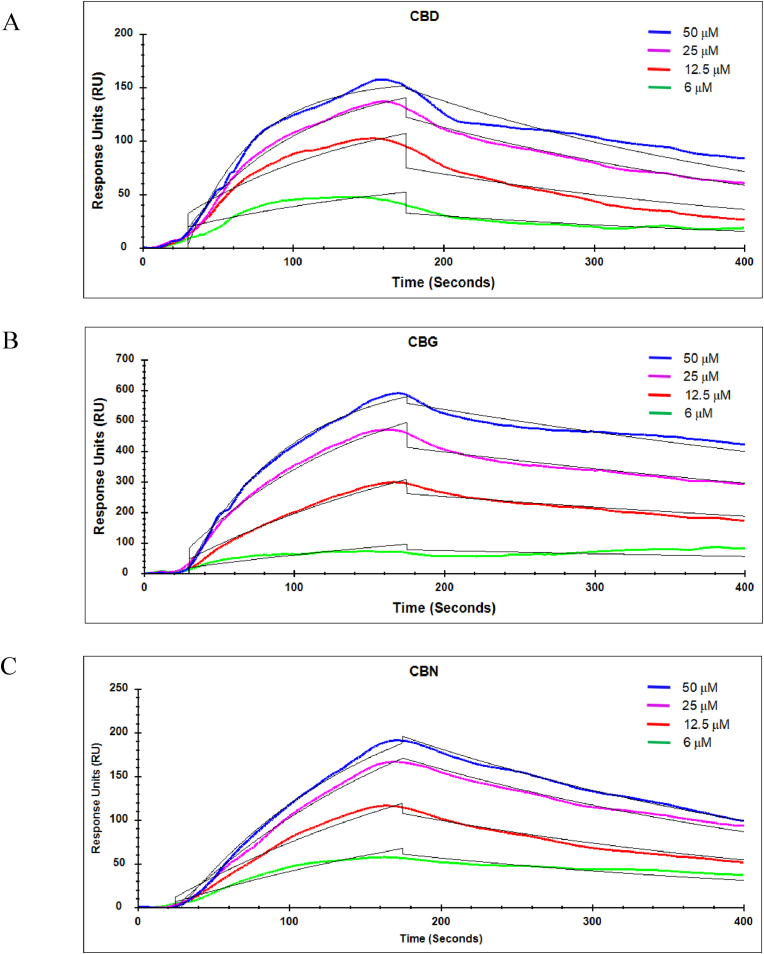
SPR analysis of CBD, CBG, and CBN binding to immobilized EGFR-TK determined using SPR measurements and analyzed with TraceDrawer 1.9.1 software (Ridgeview Instruments). SPR measurements of 2.5 ​min antigen association and 5 ​min dissociation at 25 ​°C for: CBD (A), CBG (B), and CBN (C). Curves were fitted using non-linear least squares regression with the 1:1 Langmuir binding model (theoretical sensorgrams in black). The values are expressed in mean ​± ​SEM, and the data are representative of three independent experiments. Colored lines represent the binding response signals at different antigen concentrations (50 ​μM, blue; 25 ​μM, purple; 12.5 ​μM, red; 6.25 ​μM, green; and 3.125 ​μM, sky blue), and overlaid black lines represent fitted curves. (For interpretation of the references to color in this figure legend, the reader is referred to the Web version of this article.)

To investigate the binding behavior of the cannabinoids, different concentrations of the compounds (0–50 ​μM) were injected into the flow channel and then passed over the immobilized EGFR-TK. As shown in Fig. 3, the quantitative binding kinetics were extracted from the SPR image sequences and plotted as sensorgrams. The mean dissociation constant (K_D_) value was determined from the fits to at least three independent, normalized binding curves, calculated using the Trace Drawer 1.9.1 software (Ridgeview Instruments, Sweden). CBD, CBG, and CBN bound to the immobilized EGFR-TK with K_D_ values of 8.35, 5.6, and 32.1 ​μM, respectively (Table 2). These results indicated that CBD and CBG have a higher binding affinity for EGFR-TK than CBN, being higher by about 4-fold and 6-fold, respectively, than the observed K_D_ of CBN.

**Table 2:** Binding constants between cannabinoids and EGFR-TK determined from SPR kinetic analysis.

Analyte	k_a_ (10^2^/(M∗s))	k_d_ (10^−3^/s)	K_D_ (μM)
CBD	3.92 (±2.06)	3.28 (±5.24 ​× ​10^−3^)	8.35 (±6.10)
CBG	2.64 (±0.69)	1.48 (±4.80 ​× ​10^−3^)	5.61 (±1.61)
CBN	0.94 (±0.24)	3.02 (±1.31 ​× ​10^−3^)	32.1 (±8.85)

Values are mean ​± ​SD.

### 3.3 Structural models and molecular interactions of EGFR-TK/cannabinoid complexes

**Fig. 4:**
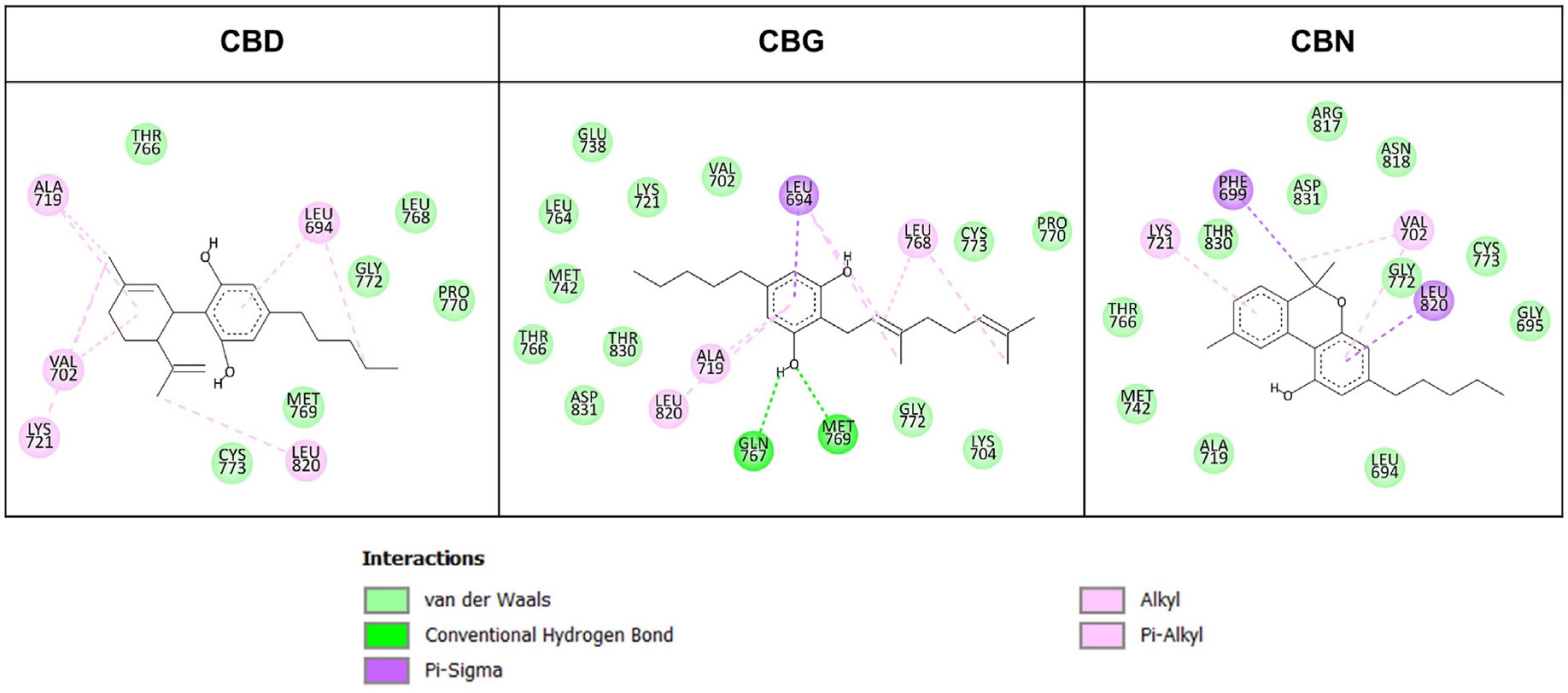
Two-dimensional illustrations of protein-ligand intermolecular interactions observed from 80 to 100 ns time averaged-simulated EGFR-TK/cannabinoid complexes. Each amino acid residue has a colored circle according to its kind of interactions.

A direct interaction of the three cannabinoids on EGFR-TK was predicted via molecular docking. The PDB code 1M17 (the active conformation of the enzyme bound with the FDA-approved drug Erlotinib) was used as the search model for a direct comparison with our previous studies (Jiwacharoenchai et al., 2022a; Jiwacharoenchai et al., 2022b). Our docking results demonstrated that the three cannabinoids were able to dock into the binding site of quinazoline-based compounds, such as Erlotinib (Fig. S1), which is the cleft between the N- and C-lobes of EGFR-TK. Structural refinements of the enzyme-inhibitor complexes via energy minimization showed that the cannabinoids shared common interactions, as observed in the main interaction of the Erlotinib binding (Stamos et al., 2002); these include the strong hydrogen bonds between a hydroxyl group of compounds and Thr766, Gln767, and Met769 of EGFR-TK, and also their non-polar moieties interacting with several amino acid residues, such as Leu694, Ala719, Lys721, Met742, Leu764, and Leu820 of the enzyme (Fig. 4, upper panel). These might indicate that the inhibition mechanism of the cannabinoids to EGFR-TK was similar to its quinazoline-based inhibitors. Molecular dynamics simulations of EGFR-TK/cannabidiol complexes revealed that the compounds remained stably bound to their binding site throughout the simulation. Backbone root mean square deviation (RMSD) of EGFR-TK in each complex showed that the enzyme structures reached equilibrium after 30 ns and fluctuated around 0.1 ​nm until the end of the simulation (Fig. S2 A). Among the three cannabinoids, heavy-atom RMSD values over the simulations exhibited the following trend in structural deviations from their initial conformation: CBD ​» ​CBN ​> ​CBG (Fig.S2 B). Visualizing the structures extracted from simulations (minimized-docked versus average-simulated structures) was consistent with the data from the RMSD calculations in that CBD and CBN explicitly exhibited a bit translation from their initial binding site, while CBG seemed to be more motionless (Fig. S3). These results suggested different mechanisms among the three cannabinoids to inhibit the function of EGFR-TK.

Binding free energies and the energetic favorableness of each cannabinoid on the binding site of EGFR-TK were estimated on the complex structures extracted from the simulations (Table 3). We found that relaxing the docked complexes via a molecular dynamics simulation, showed that all three EGFR-TK/cannabinoid complexes still had large negative amounts of binding free energy; ΔGbind average from t30 to t50 ns was ≈ −89.4, −83.5, and −90.6 ​kJ/mol, while the average from t80 to t100 ns was ≈ −99.2, −106.5, and −85.5 ​kJ/mol for CBD, CBG and CBN, respectively. We also found that the binding energetics of the three cannabinoids were more dominated by van der Waals (vdW) over electrostatic interactions, suggesting an energy preference of the compounds for binding to EGFR-TK. It should be noted that these estimations of ΔGbind average from t80 to t100 ns were consistent with our *in vitro* kinase inhibitory activity and SPR analyses, in which the trend in ligand binding affinity was CBG ​> ​CBD ​> ​CBN. Nevertheless, the binding strength of the three cannabinoids on EGFR-TK seemed to be weaker than the Erlotinib binding, since the drug had a lower binding free energy than the cannabinoids (Jiwacharoenchai et al., 2022).

**Table 3:** Comparison of binding free energy components estimated from minimized-docked and average-simulated EGFR-TK/cannabinoid complexes.

Energy term	Minimized-docked complex			Average simulated complex (30–50 ns)			Average simulated complex (80–100 ns)		
	CBD	CBG	CBN	CBD	CBG	CBN	CBD	CBG	CBN
Δ*E*_vdW_	−190.6	−185.9	−181.3	−135.1 ​± ​11.8	−160.3 ​± ​13.2	−164.6 ​± ​12.9	−126.2 ​± ​11.4	−167.0 ​± ​9.8	−156.7 ​± ​10.6
Δ*E*_elec_	−19.0	−22.9	−25.0	−13.3 ​± ​4.7	−17.2 ​± ​6.9	−17.2 ​± ​5.6	−10.2 ​± ​3.5	−21.0 ​± ​8.9	−15.1 ​± ​5.2
Δ*G*_polar_	115.5	102.5	126.5	75.6 ​± ​20.7	113.4 ​± ​14.5	110.6 ​± ​15.3	53.3 ​± ​8.8	100.0 ​± ​11.2	105.6 ​± ​15.2
Δ*G*_nonpolar_	−18.6	−19.9	−18.4	−16.6 ​± ​1.7	−19.5 ​± ​1.1	−19.5 ​± ​1.0	−16.1 ​± ​1.0	−18.5 ​± ​0.9	−19.2 ​± ​1.0
Δ*G*_bind_	−112.7	−126.1	−98.2	−89.4 ​± ​21.1	−83.5 ​± ​16.1	−90.6 ​± ​17.2	−99.2 ​± ​11.4	−106.5 ​± ​12.6	−85.5 ​± ​12.0

Values are mean ​± ​SD.

**Fig. 5:**
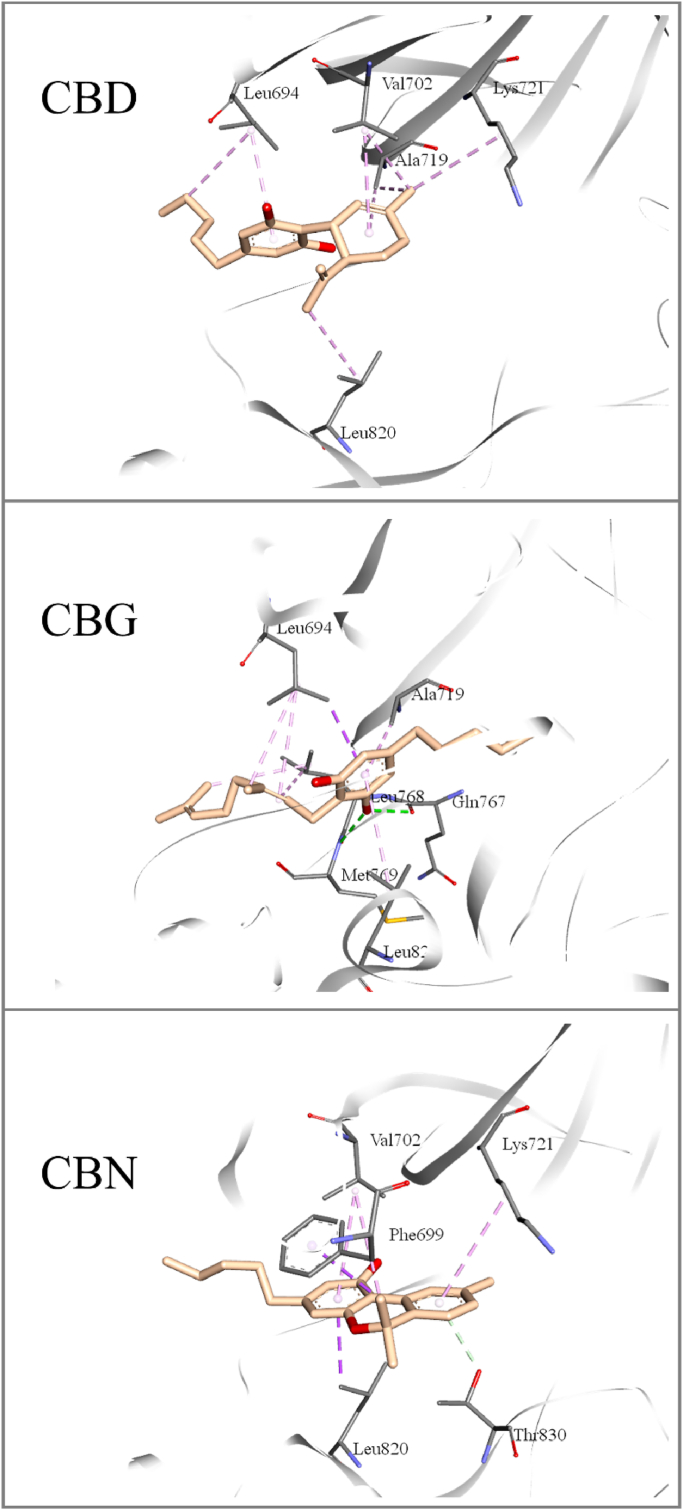
Three-dimensional illustrations of protein-ligand intermolecular interactions between observed from average-simulated (t80-t100 ns) EGFR-TK/cannabinoid complexes. Non-polar and polar interactions are represented in pink and green dotted lines, respectively. (For interpretation of the references to color in this figure legend, the reader is referred to the Web version of this article.)

Intermolecular interactions between the amino acid residues located in the EGFR-TK binding site and three cannabinoids were identified from the average simulated structures and are illustrated in Figs. 4 and 5, and Fig. S4. The numbers of such interactions seemed to be higher in EGFR-TK/CBG than in the EGFR-TK/CBD and EGFR-TK/CBN complexes. We found several kinds of non-polar contacts (vdW, alkyl-alkyl and pi-alkyl interactions), where most contributed to the EGFR-TK/cannabinoid complexes. Additionally, hydrogen bonding was observed between one hydroxyl group of CBG with the main chain of Met769 (Figs. 4 and 5). This interaction would have a remarkable influence on the EGFR-TK/CBG complex, since a hydrogen bond between the hinge region residue Met769 of EGFT-TK and the adenine moiety of quinazoline inhibitors has been known to be the crucial interaction (Bhullar et al., 2018). No substantial hydrogen bond was observed in the simulation of the EGFR-TK/CBD and EGFR-TK/CBN complexes. Taken together, our simulations suggested differences in favorable interactions of the three cannabinoids with EGFR-TK, which eventually led to their different levels of inhibitory activity and binding affinity against the enzyme.

### 3.4 Anticancer activity of cannabinoids on EGFR-positive cancer cells

**Fig. 6:**
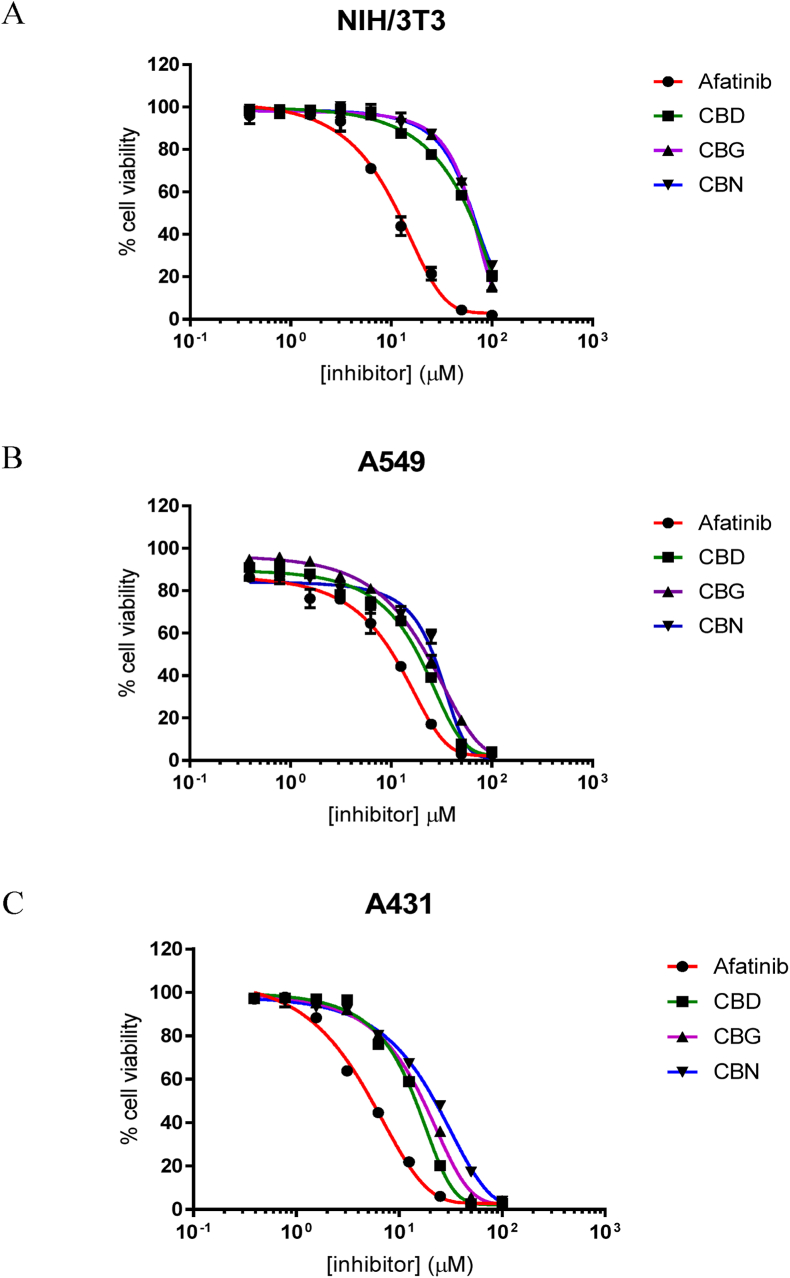
Growth inhibitory effects of Afatinib and cannabinoids treatments on: A) NIH/3T3 (mouse fibroblast (non-tumor cells)), B) A549 (EGFR-positive human lung cancer), and C) A431 (EGFR-positive epidermoid carcinoma). IC_50_ values are shown in Table 1, results are expressed as mean ​± ​SD of triplicate experiments.

All compounds were evaluated for anticancer activity against NIH/3T3, A549, and A431. Following 72 ​h of continuous exposure to the cannabinoids or Afatinib, all compounds were very sensitive to EGFR-positive cells and showed a dose-dependent manner (Fig. 6). Among the cell lines, CBD had the lowest IC_50_ compared to the others both in A431 (14.18 ​μM) and A549 (22.44 ​μM), while the NIH/3T3 cells line had a lower sensitivity to all compounds (Table 1).

### 3.5 Apoptosis induction of cannabinoids in EGFR-positive cancer cells A431

**Fig. 7:**
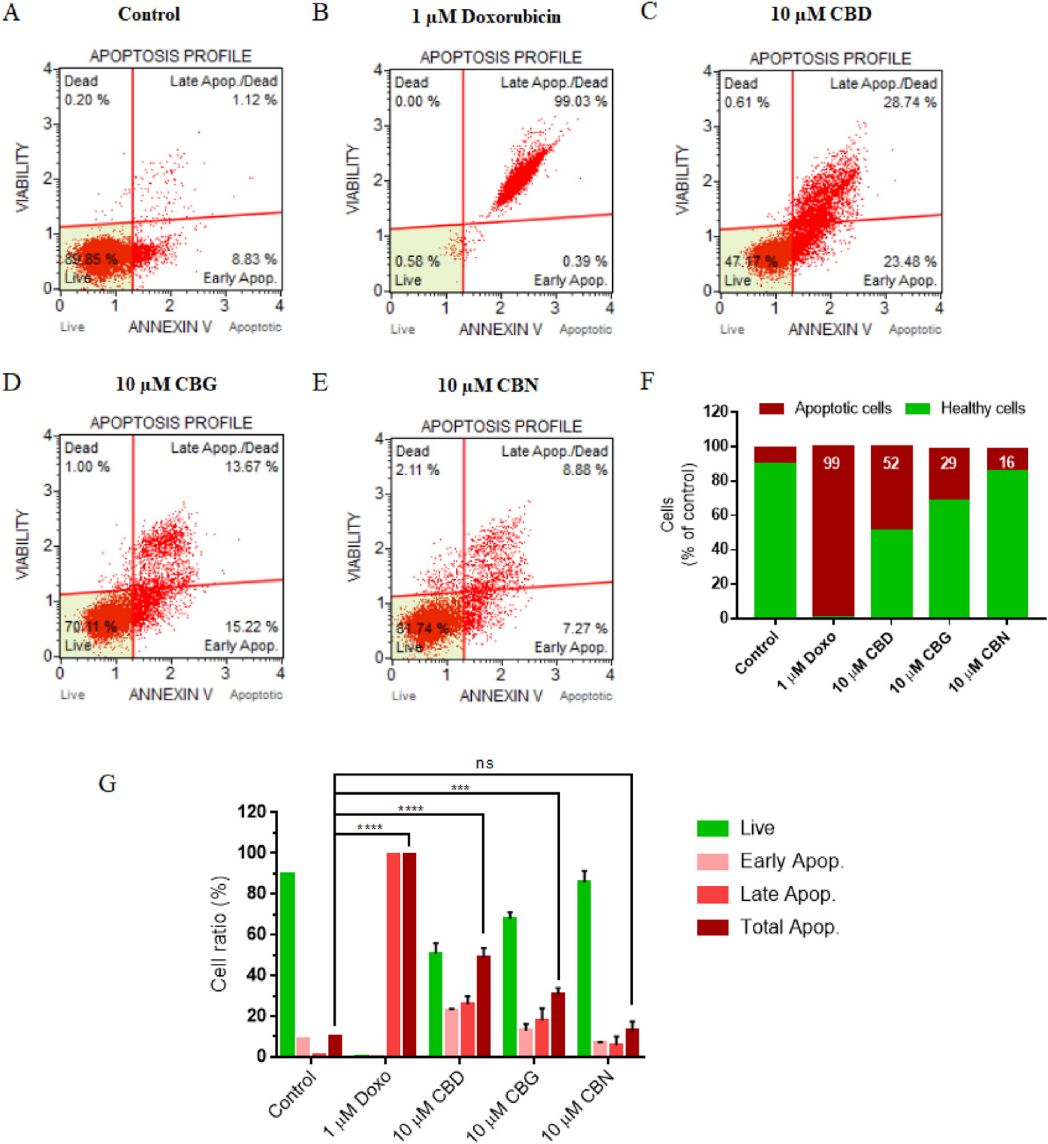
Effect of cannabinoids on A431 ​cell apoptosis. (A–E) Apoptosis profile (Muse ™ Annexin V & Dead Cell Assay) demonstrating that proportion of total apoptotic cells increased in A431 ​cells after treatment with 1 ​μM doxorubicin (positive control) and 10 ​μM cannabinoids (CBD, CBG, and CBN) compared with untreated control. In all cases, flow cytometric analysis was determined at 72 ​h after treatment. Each plot has 4 quadrant markers, reflecting different cellular states: upper left quadrant ​= ​dead cells (necrosis), upper right ​= ​late apoptotic/dead cells (cells that are positive for both Annexin V and for cell death marker 7-AAD, 7-Aminoactinomycin D), lower left ​= ​live cells, and lower right ​= ​early apoptotic cells (cells that are positive only for Annexin V). Triplicate experiments were conducted and representative results are shown. (F) Bar graph data of apoptosis and healthy cell ratio. (G) Percentage of live cells, cells in early apoptosis, cells in late apoptosis or total apoptosis. Total apoptotic cells significantly increased in A431 ​cells treated with CBD (∗∗∗∗P ​< ​0.0001) and CBG (∗∗∗P ​= ​0.008), compared with control group. Triplicate samples used to obtain mean ​± ​SD shown by error bars.

An Annexin V assay was performed in A431 ​cells to assess the proapoptotic effect of cannabinoids. The representative flow cytometry plots showed proportions of four groups of A431 ​cells after the different treatments (Fig. 7A−F). A significant increase in the number of apoptotic cells from 9.95% to 52.22% and to 28.89% after treatment with CBD and CBG, respectively, was observed compared to the control group (Fig. 7F). The ratios of A431 ​cells at each stage of apoptosis after treatment with 1 ​μM doxorubicin (positive control) and 10 ​μM of cannabinoids (CBD, CBG, and CBN), are summarized in Fig. 7G. Among the cannabinoids, CBD induced the highest total apoptosis with 52.22% (23.48% early apoptosis and 28.74% late apoptosis) compared to 28.89% for the CBG and 16.15% for the CBN treatments. These results indicated that most of the antiproliferative activity of the cannabinoids in A431 ​cells might have been mediated by apoptosis.

## 4 Discussion

The present study investigated the binding affinity and inhibitory properties of CBD, CBG, and CBN on EGFR-TK. Among these compounds, CBD and CBG showed potent inhibitory activity against EGFR-TK, while the inhibitory activity of CBN was weaker. CBD and CBG were more active than CBN on EGFR-tyrosine kinase inhibition. Additionally, binding affinities, based on K_D_ values, were determined using SPR and indicated the high binding affinity of CBD and CBG to the immobilized EGFR-TK, while CBN had a relatively lower binding affinity compared to CBD and CBG. The molecular structures of these compounds contain aromatic rings and hydroxyl groups (Fig. 1), which are important for forming hydrophobic interaction and hydrogen bonds with many amino acids in the EGFR active site; there are two hydroxyl groups on CBD and CBG, whereas CBN has only 1 group (Morales et al., 2017). The correlation between these two results indicated the importance of the phenolic hydroxyl groups in these cannabinoids for both binding and inhibition of EGFR-TK. According to the results of the molecular docking and dynamics simulations, the three cannabinoids could be stably docked into the ATP-binding site of EGFR-TK through multiple weak interactions, including the hydrophobic interaction among several amino acid residues, strong hydrogen bonds between a hydroxyl group of cannabinoids and Thr766, Gln767, and Met769 of EGFR-TK, and van der Waals interactions. For the inhibition of several receptor tyrosine kinases, 4-anilinoquinazolines have emerged as a versatile template. Among them, small molecule EGFR-TKIs, such as Gefitinib and Erlotinib, were introduced for cancer treatment (Sadek et al., 2014). Generally, the chemical structures of most TKIs include single or multiple aromatic groups with carboxyl or hydroxyl, amino, and in some cases, methyl or ether moieties (Singh et al., 2015). An inhibitor's ability to access the hydrophobic pocket of the ATP-competitive site depends on the position of the hydrophobic interaction force between the candidate compounds and the EGFR-TK active site. (Choowongkomon et al., 2010). The three tested cannabinoids were potent growth inhibitors and showed a dose-dependent inhibition in the EGFR-positive epidermoid carcinoma A431 and lung cancer A549 ​cell lines. Among these compounds, CBD had the most anticancer activity followed by CBG and CBN, respectively. The results had similar trends as those for A431 and A549 ​cells, but there was a very low cytotoxic effect in the cannabinoids treatment in NIH/3T3 normal cells. However, these compounds are more sensitive to A431 than A549, so it is possible that the cytotoxicity varies with the quantification of EGFR expression level. The averaged A431 ​cell surface EGFR density was found to be 636 receptors/μm^2^ with an estimation of 5 ​× ​10^5^ EGFR per cell, while the EGFR density in A549 was 142 receptors/μm^2^, corresponding to 1.1 ​× ​10^5^ per cell and much lower than for A431 (Zhang et al., 2015). Furthermore, significant increases in the numbers of apoptotic cells in A431 from 9.95% to 52.22% and to 28.89% after treatment with CBD and CBG, respectively, were observed compared to the control group. These results indicated that most of the antiproliferative activity of the cannabinoids in the EGFR-positive cells was mediated by apoptosis. This finding suggested that CBD and CBG could be developed for EGFR-TKIs-based treatment options in patients with EGFR-positive cancers.

## 5 Conclusion

We assessed the kinase-inhibition activity of cannabinoids (CBD, CBG, and CBN) to intracellular tyrosine-kinase of EGFR. The SPR analysis provided binding kinetics information of the cannabinoids on EGFR-TK. CBD and CBG had a high binding affinity and kinase-inhibitory activity against EGFR-TK, while CBN had relatively lower values than CBD and CBG. The computational analysis further revealed that the hydrophobic interaction and hydrogen bonding between a hydroxyl group of the cannabinoids and Thr766, Gln767, and Met769 of EGFR-TK enabled the three cannabinoids to be stably docked into the active site of EGFR-TK. All three cannabinoids had high levels of inhibition in EGFR-positive cancer cells, but low levels in normal cells. CBD was more effective in suppressing cancer cells and inducing apoptosis than the other compounds, while cells with higher EGFR expression were more likely to be affected by cannabinoid-induced cytotoxicity. Furthermore, CBD and CBG significantly induced cell apoptosis in the EGFR-positive cancer A431 line. The consistency between the *in vitro* and *in silico* studies suggested that the phenolic hydroxyl group is essential for the anticancer activity of the cannabinoids. However, a combined computational approach, signaling pathway analysis, structural analysis, and *in vivo* studies will be required for the further investigation of molecular interactions and biological mechanisms. These findings demonstrate that the cannabinoids could be transformed into unique natural compounds for use in the development of anti-EGFR-positive cancer therapies.

## Funding

This research was funded by the Royal Golden Jubilee PhD Program (grant number PHD/0152/2557), 10.13039/501100004704National Research Council of Thailand (NRCT), Thailand and by the 10.13039/501100005621Kasetsart University Research and Development Institute (KURDI) (grant ID: FF (KU) 6.64), Kasetsart University, Thailand.

## CRediT authorship contribution statement

**Thomanai Lamtha:** All authors conceived and designed the experiments, carried out the experiments, contributed to the interpretation of the results. **Lueacha Tabtimmai:** All authors conceived and designed the experiments. **Napat Songtawee:** All authors conceived and designed the experiments, carried out the experiments, contributed to the interpretation of the results. **Natthasit Tansakul:** Writing – review &; editing, with support. **Kiattawee Choowongkomon:** carried out the experiments, Writing – review &; editing, with support, All authors discussed the results and contributed to the final manuscript, Supervision.

## Declaration of competing interest

The authors declare that they have no known competing financial interests or personal relationships that could have appeared to influence the work reported in this paper.

## References

[bib1] Baek S.H., Kim Y.O., Kwag J.S., Choi K.E., Jung W.Y., Han D.S. (1998). Boron trifluoride etherate on silica-A modified Lewis acid reagent (VII). Antitumor activity of cannabigerol against human oral epitheloid carcinoma cells. Arch Pharm. Res. (Seoul).

[bib2] Baker N.A., Sept D., Joseph S., Holst M.J., McCammon J.A. (2001). Electrostatics of nanosystems: application to microtubules and the ribosome. Proc. Natl. Acad. Sci. U. S. A.

[bib3] Bhullar K.S., Lagarón N.O., McGowan E.M., Parmar I., Jha A., Hubbard B.P., Rupasinghe H.P.V. (2018). Kinase-targeted cancer therapies: progress, challenges and future directions. Mol. Cancer.

[bib4] Block E.R., Klarlund J.K. (2008). Wounding sheets of epithelial cells activates the epidermal growth factor receptor through distinct short- and long-range mechanisms. Mol. Biol. Cell.

[bib5] Borrelli F., Pagano E., Romano B., Panzera S., Maiello F., Coppola D., de Petrocellis L., Buono L., Orlando P., Izzo A.A. (2014). Colon carcinogenesis is inhibited by the TRPM8 antagonist cannabigerol, a Cannabis-derived non-psychotropic cannabinoid. Carcinogenesis.

[bib6] Bose R. (2013). A neu view of invasive lobular breast cancer. Clin. Cancer Res..

[bib7] Bussi G., Donadio D., Parrinello M. (2007). Canonical sampling through velocity rescaling. J. Chem. Phys..

[bib8] Cai W.-Q., Zeng L.-S., Wang L.-F., Wang Y.-Y., Cheng J.-T., Zhang Y., Han Z.-W., Zhou Y., Huang S.-L., Wang X.-W., Peng X.-C., Xiang Y., Ma Z., Cui S.-Z., Xin H.-W. (2020). The latest battles between EGFR monoclonal antibodies and resistant tumor cells. Front. Oncol..

[bib9] Cascio M.G., Gauson L.A., Stevenson L.A., Ross R.A., Pertwee R.G. (2010). Evidence that the plant cannabinoid cannabigerol is a highly potent alpha2-adrenoceptor agonist and moderately potent 5HT1A receptor antagonist. Br. J. Pharmacol..

[bib10] Choowongkomon K., Sawatdichaikul O., Songtawee N., Limtrakul J. (2010). Receptor-based virtual screening of EGFR kinase inhibitors from the NCI diversity database. Molecules.

[bib11] Cole J.C., Murray C.W., Nissink J.W.M., Taylor R.D., Taylor R. (2005). Comparing protein-ligand docking programs is difficult. Proteins.

[bib12] Cullen W., Turega S., Hunter C.A., Ward M.D. (2015). Virtual screening for high affinity guests for synthetic supramolecular receptors. Chem. Sci..

[bib13] Darden T., York D., Pedersen L. (1993). Particle mesh Ewald: an N⋅log(N) method for Ewald sums in large systems. J. Chem. Phys..

[bib14] de Petrocellis L., Ligresti A., Moriello A.S., Allarà M., Bisogno T., Petrosino S., Stott C.G., di Marzo V. (2011). Effects of cannabinoids and cannabinoid-enriched Cannabis extracts on TRP channels and endocannabinoid metabolic enzymes. Br. J. Pharmacol..

[bib15] de Petrocellis L., Orlando P., Moriello A.S., Aviello G., Stott C., Izzo A.A., di Marzo V. (2012). Cannabinoid actions at TRPV channels: effects on TRPV3 and TRPV4 and their potential relevance to gastrointestinal inflammation. Acta Physiologica (Oxford, England).

[bib16] Elbaz M., Nasser M.W., Ravi J., Wani N.A., Ahirwar D.K., Zhao H., Oghumu S., Satoskar A.R., Shilo K., Carson W.E., Ganju R.K. (2015). Modulation of the tumor microenvironment and inhibition of EGF/EGFR pathway: novel anti-tumor mechanisms of Cannabidiol in breast cancer. Molecular Oncology.

[bib17] Hess B., Bekker H., Berendsen H.J.C., Fraaije J.G.E.M. (1997). LINCS: a linear constraint solver for molecular simulations. J. Comput. Chem..

[bib18] Jakalian A., Jack D.B., Bayly C.I. (2002). Fast, efficient generation of high-quality atomic charges. AM1-BCC model: II. Parameterization and validation. J. Comput. Chem..

[bib19] Jiwacharoenchai N., Saruengkhanphasit R., Niwetmarin W., Seetaha S., Choowongkomon K., Ruchirawat S., Eurtivong C. (2022). Discovery of potent antiproliferative agents from selected oxygen heterocycles as EGFR tyrosine kinase inhibitors from the U.S. National Cancer Institute database by in silico screening and bioactivity evaluation. Bioorg. Med. Chem. Lett.

[bib20] Jiwacharoenchai N., Tabtimmai L., Kiriwan D., Suwattanasophon C., Seetaha S., Sinthuvanich C., Choowongkomon K. (2022). A novel cyclic NP1 reveals obstruction of EGFR kinase activity and attenuation of EGFR-driven cell lines. J. Cell. Biochem..

[bib21] Jones G., Willett P., Glen R.C., Leach A.R., Taylor R. (1997). Development and validation of a genetic algorithm for flexible docking. J. Mol. Biol..

[bib22] Jorgensen W.L., Chandrasekhar J., Madura J.D., Impey R.W., Klein M.L. (1983). Comparison of simple potential functions for simulating liquid water. J. Chem. Phys..

[bib23] Kumagai S., Koyama S., Nishikawa H. (2021). Antitumour immunity regulated by aberrant ERBB family signalling. Nat. Rev. Cancer.

[bib24] Kumari R., Kumar R., Lynn A. (2014). g_mmpbsa--a GROMACS tool for high-throughput MM-PBSA calculations. J. Chem. Inf. Model..

[bib25] Ligresti A., Moriello A.S., Starowicz K., Matias I., Pisanti S., de Petrocellis L., Laezza C., Portella G., Bifulco M., di Marzo V. (2006). Antitumor activity of plant cannabinoids with emphasis on the effect of cannabidiol on human breast carcinoma. J. Pharmacol. Exp. Therapeut..

[bib26] Massi P., Solinas M., Cinquina V., Parolaro D. (2013). Cannabidiol as potential anticancer drug. Br. J. Clin. Pharmacol..

[bib27] Morales P., Reggio P.H., Jagerovic N. (2017). https://www.frontiersin.org/article/10.3389/fphar.2017.00422.

[bib28] Nan X., Xie C., Yu X., Liu J. (2017). EGFR TKI as first-line treatment for patients with advanced EGFR mutation-positive non-small-cell lung cancer. Oncotarget.

[bib29] Nosé S., Klein M.L. (1983). Constant pressure molecular dynamics for molecular systems. Mol. Phys..

[bib30] Oguntibeju O.O. (2019). Medicinal plants and their effects on diabetic wound healing. Vet. World.

[bib31] Pedrazzi J.F.C., Sales A.J., Guimarães F.S., Joca S.R.L., Crippa J.A.S., del Bel E. (2021). Cannabidiol prevents disruptions in sensorimotor gating induced by psychotomimetic drugs that last for 24-h with probable involvement of epigenetic changes in the ventral striatum. Prog. Neuro Psychopharmacol. Biol. Psychiatr..

[bib32] Pucci M., Rapino C., di Francesco A., Dainese E., D'Addario C., Maccarrone M. (2013). Epigenetic control of skin differentiation genes by phytocannabinoids. Br. J. Pharmacol..

[bib33] Rong C., Lee Y., Carmona N.E., Cha D.S., Ragguett R.-M., Rosenblat J.D., Mansur R.B., Ho R.C., McIntyre R.S. (2017). Cannabidiol in medical marijuana: research vistas and potential opportunities. Pharmacol. Res..

[bib34] Roskoski R. (2004). The ErbB/HER receptor protein-tyrosine kinases and cancer. Biochem. Biophys. Res. Commun..

[bib35] Russo E.B., Marcu J. (2017). Cannabis pharmacology: the usual suspects and a few promising leads. Advances in Pharmacology (San Diego, Calif.).

[bib36] Sadek M.M., Serrya R.A., Kafafy A.-H.N., Ahmed M., Wang F., Abouzid K.A.M. (2014). Discovery of new HER2/EGFR dual kinase inhibitors based on the anilinoquinazoline scaffold as potential anti-cancer agents. J. Enzym. Inhib. Med. Chem..

[bib37] Sawatdichaikul O., Hannongbua S., Sangma C., Wolschann P., Choowongkomon K. (2012). *In silico* screening of epidermal growth factor receptor (EGFR) in the tyrosine kinase domain through a medicinal plant compound database. J. Mol. Model..

[bib38] Schoeman R., Beukes N., Frost C. (2020). Cannabinoid combination induces cytoplasmic vacuolation in MCF-7 breast cancer cells. Molecules (Basel, Switzerland).

[bib39] Shrivastava A., Kuzontkoski P.M., Groopman J.E., Prasad A. (2011). Cannabidiol induces programmed cell death in breast cancer cells by coordinating the cross-talk between apoptosis and autophagy. Mol. Cancer Therapeut..

[bib40] Singh H., Singh S., Singla D., Agarwal S.M., Raghava G.P.S. (2015). QSAR based model for discriminating EGFR inhibitors and non-inhibitors using Random forest. Biol. Direct.

[bib41] Soroceanu L., Murase R., Limbad C., Singer E., Allison J., Adrados I., Kawamura R., Pakdel A., Fukuyo Y., Nguyen D., Khan S., Arauz R., Yount G.L., Moore D.H., Desprez P.-Y., McAllister S.D. (2013). Id-1 is a key transcriptional regulator of glioblastoma aggressiveness and a novel therapeutic target. Cancer Res..

[bib42] Sousa da Silva A.W., Vranken W.F. (2012). Acpype - AnteChamber PYthon parser interfacE. BMC Res. Notes.

[bib43] Spix J.K., Chay E.Y., Block E.R., Klarlund J.K. (2007). Hepatocyte growth factor induces epithelial cell motility through transactivation of the epidermal growth factor receptor. Exp. Cell Res..

[bib44] Stamos J., Sliwkowski M.X., Eigenbrot C. (2002). Structure of the epidermal growth factor receptor kinase domain alone and in complex with a 4-anilinoquinazoline inhibitor. J. Biol. Chem..

[bib45] Stefkov G., Cvetkovikj Karanfilova I., Stoilkovska Gjorgievska V., Trajkovska A., Geskovski N., Karapandzova M., Kulevanova S. (2022). Analytical techniques for phytocannabinoid profiling of cannabis and cannabis-based products-A comprehensive review. Molecules (Basel, Switzerland).

[bib56] Stierand K., Rarey M. (2010). PoseView -- molecular interaction patterns at a glance. J. Cheminf..

[bib46] Thomson R.J., Moshirfar M., Ronquillo Y. (2022).

[bib47] Turke A.B., Song Y., Costa C., Cook R., Arteaga C.L., Asara J.M., Engelman J.A. (2012). MEK inhibition leads to PI3K/AKT activation by relieving a negative feedback on ERBB receptors. Cancer Res..

[bib48] van der Spoel D., Lindahl E., Hess B., Groenhof G., Mark A.E., Berendsen H.J.C. (2005). GROMACS: fast, flexible, and free. J. Comput. Chem..

[bib49] Verdonk M.L., Cole J.C., Hartshorn M.J., Murray C.W., Taylor R.D. (2003). Improved protein-ligand docking using GOLD. Proteins.

[bib50] Vrechi T.A.M., Leão A.H.F.F., Morais I.B.M., Abílio V.C., Zuardi A.W., Hallak J.E.C., Crippa J.A., Bincoletto C., Ureshino R.P., Smaili S.S., Pereira G.J.S. (2021). Cannabidiol induces autophagy via ERK1/2 activation in neural cells. Sci. Rep..

[bib51] Wilkinson J.D., Williamson E.M. (2007). Cannabinoids inhibit human keratinocyte proliferation through a non-CB1/CB2 mechanism and have a potential therapeutic value in the treatment of psoriasis. J. Dermatol. Sci..

[bib52] Yang H., Wang Z., Capó-Aponte J.E., Zhang F., Pan Z., Reinach P.S. (2010). Epidermal growth factor receptor transactivation by the cannabinoid receptor (CB1) and transient receptor potential vanilloid 1 (TRPV1) induces differential responses in corneal epithelial cells. Exp. Eye Res..

[bib53] Yarden Y., Sliwkowski M.X. (2001). Untangling the ErbB signalling network. Nat. Rev. Mol. Cell Biol..

[bib54] Yu D., Hung M.C. (2000). Overexpression of ErbB2 in cancer and ErbB2-targeting strategies. Oncogene.

[bib55] Zhang F., Wang S., Yin L., Yang Y., Guan Y., Wang W., Xu H., Tao N. (2015). Quantification of epidermal growth factor receptor expression level and binding kinetics on cell surfaces by surface plasmon resonance imaging. Anal. Chem..

