# Supplementary material for: Structural analysis of cannabinoids against EGFR-TK leads a novel target against EGFR-driven cell lines
Source: Curr Res Pharmacol Drug Discov. 2022 Sep 30;3:100132. doi: 10.1016/j.crphar.2022.100132 (PMC9780064; doi:10.1016/j.crphar.2022.100132)
Supplement: Multimedia component 1 [file mmc1.docx]

Supplementary material

Structural analysis of Cannabinoids against EGFR-TK leads a novel target against EGFR-driven cell lines

Thomanai Lamtha^a,b^, Lueacha Tabtimmai^c^, Napat Songtawee^d^, Natthasit Tansakul^e^, Kiattawee Choowongkomon^a,^*

*^a^ Laboratory of Protein Engineering and Bioinformatics (PROTEB), Department of Biochemistry, Faculty of Science, Kasetsart University, Bangkok, 10900, Thailand*

*^b^ Spectroscopic and Sensing Devices Research Group (SSDRG), National Electronics and Computer Technology Center (NECTEC), National Science and Technology Development Agency (NSTDA), Pathumthani, 12120, Thailand*

*^c^ Department of Biotechnology, Faculty of Applied Science, King Mongkut’s University of Technology North Bangkok, Bangkok, 10800, Thailand*

*^d^ Department of Clinical Chemistry, Faculty of Medical Technology, Mahidol University, Nakhon Pathom, 73170, Thailand*

*^e^ Department of Pharmacology, Faculty of Veterinary Medicine, Kasetsart University, Bangkok, 10900, Thailand*

*** Corresponding author:** Kiattawee Choowongkomon

**E-mail address:** kiattawee.c@ku.th

**E-mails addresses:** thomanai.l@ku.th (Thomanai Lamtha); lueacha.t@sci.kmutnb.ac.th (Lueacha Tabtimmai); fvetnst@ku.ac.th (Natthasit Tansakul); napat.son@mahidol.ac.th (Napat Songtawee)


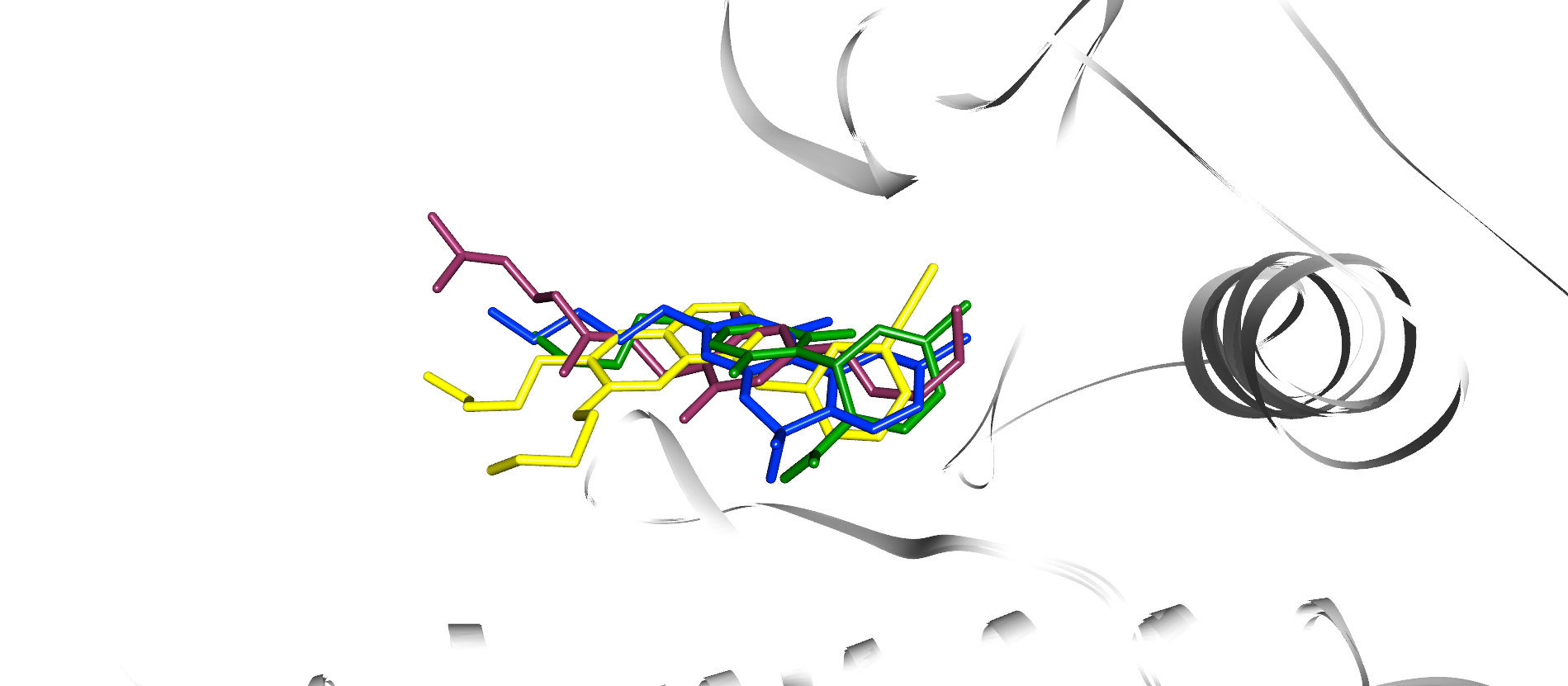


**Supplementary Figure 1:** 3D illustrations of docked conformation of CBD (green), CBG (dark pink) and CBN (blue) compared to conformation of Erlotinib (yellow) on EGFR-TK binding site. Ligand and protein structures are shown as sticks and ribbons, respectively.


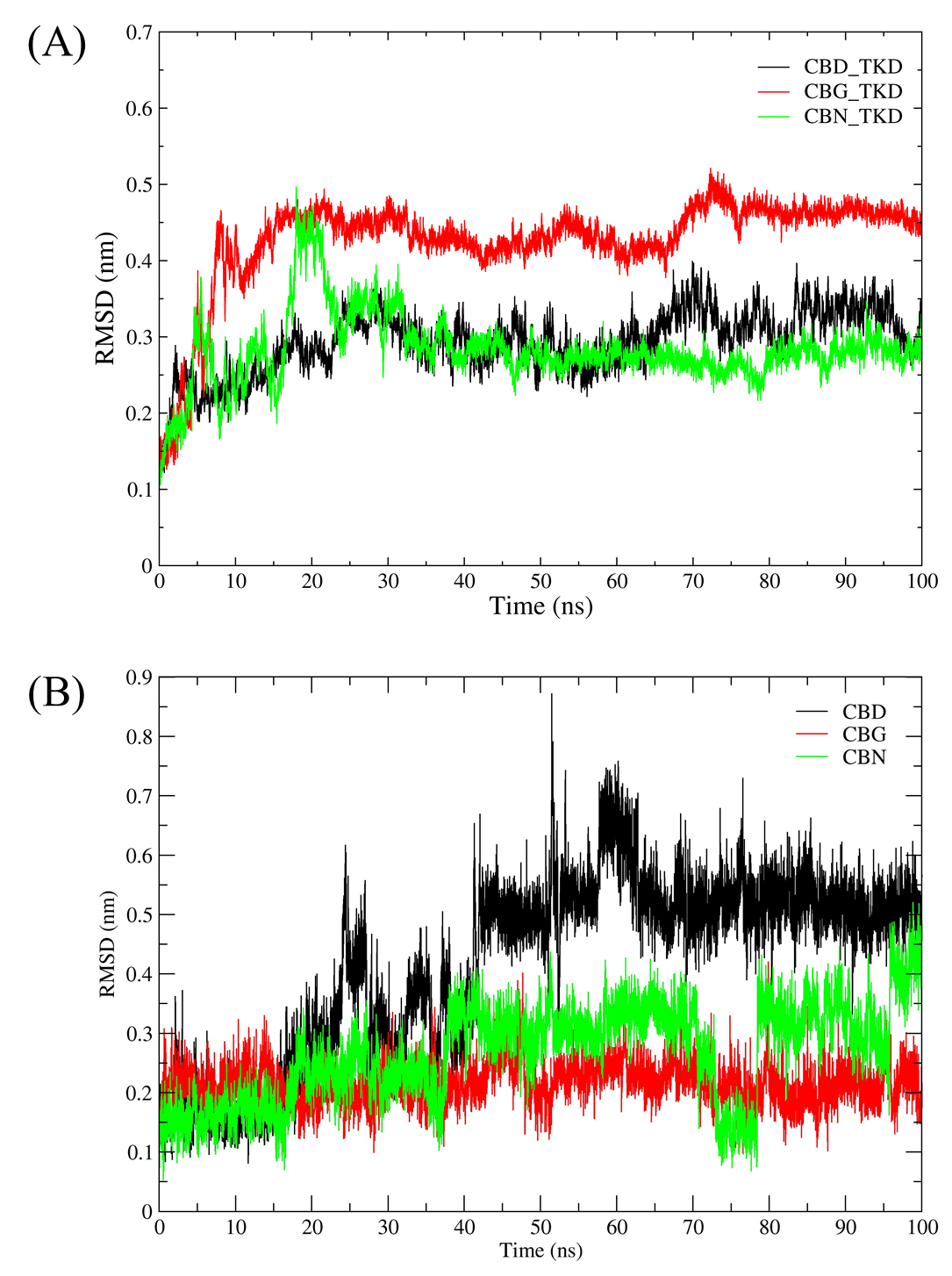


**Supplementary Figure 2:** Root mean square deviations (RMSD) of protein backbones (A) and ligand atoms (B) as function of simulation time with respect to their initial coordinates for three EGFR-TK/cannabinoid complexes.


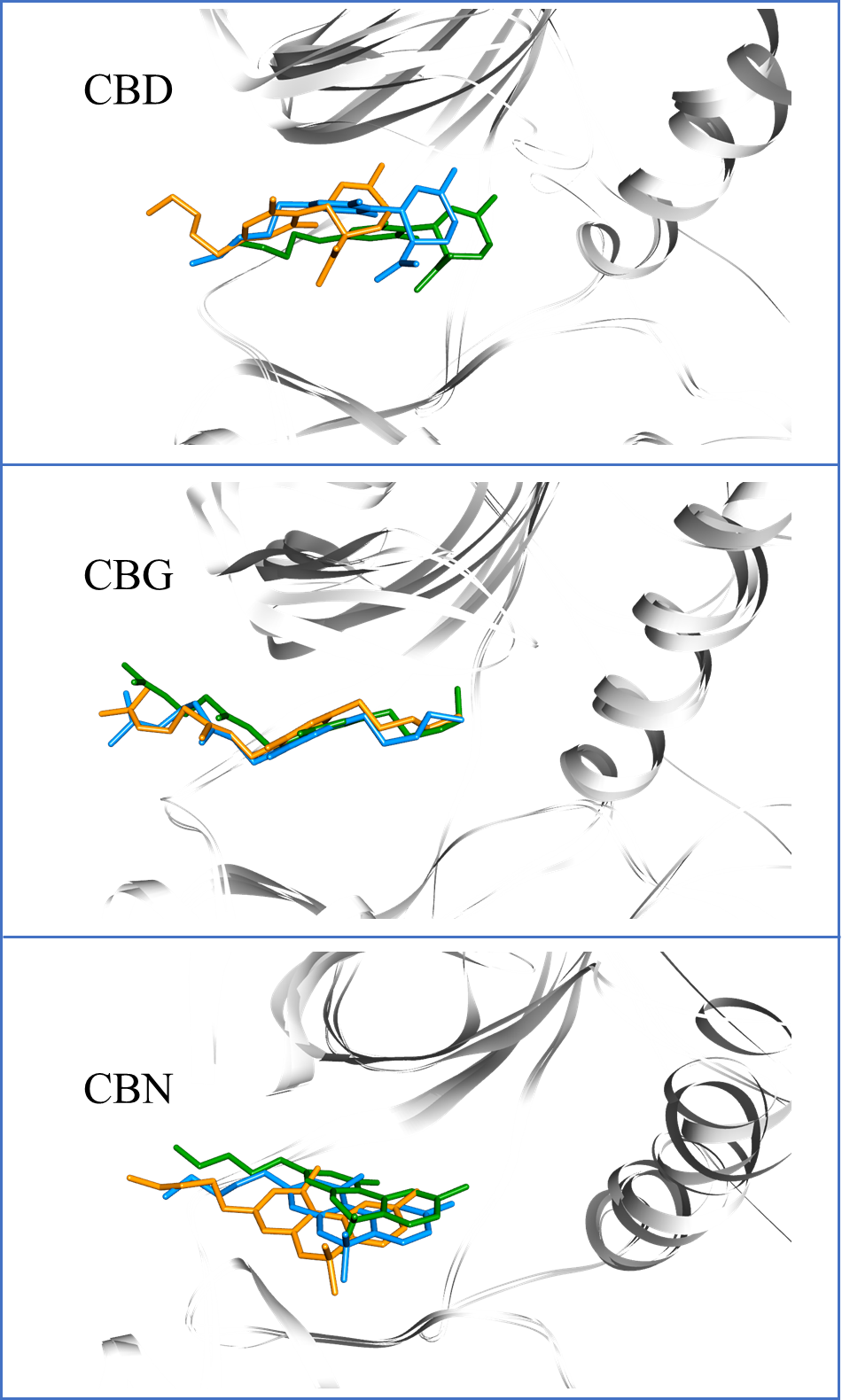


**Supplementary Figure 3:** 3D illustrations of complex structures extracted from molecular dynamics simulations; average-simulated t30–t50 ns (blue) and t80–t100 ns (orange) compared to their docked conformation (green) from CBD, CBG and CBN simulations. Ligand and protein structures are shown as sticks and ribbons, respectively.


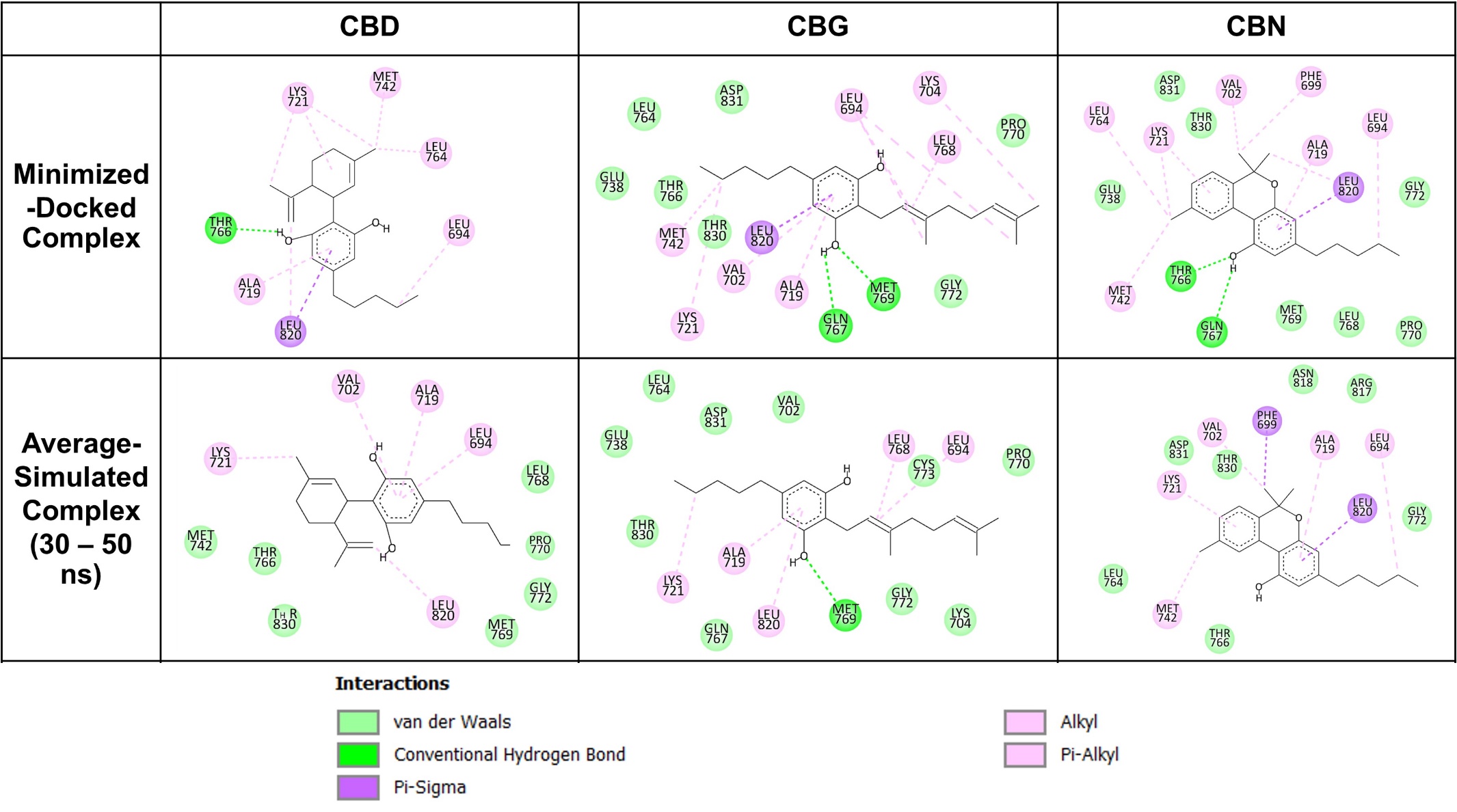


**Supplementary Figure 4:** Two-dimensional illustrations of protein-ligand intermolecular interactions between observed from minimized-docked and 30 to 50 time averaged-simulated EGFR-TK/cannabinoid complexes. Each amino acid residue has a colored circle according to its kind of interactions.
